# Cytokine Release Syndrome Associated With Immune‐Modulating Chemotherapy: Potential Mitigating Role of Intravenous Omega‐3 Fatty Acid Triglycerides

**DOI:** 10.1002/cnr2.70025

**Published:** 2024-10-07

**Authors:** David F. Driscoll, Bruce R. Bistrian

**Affiliations:** ^1^ Stable Solutions LLC Easton Massachusetts USA; ^2^ UMASS Chan Medical School Worcester Massachusetts USA; ^3^ Harvard Medical School Boston Massachusetts USA

**Keywords:** cytokine release syndrome, immune‐modulating chemotherapy, omega‐3 fatty acids

## Abstract

**Background:**

Patient susceptibility to cytokine release syndrome (CRS) resulting from immune‐modulating chemotherapy has profound implications for clinical outcome. This is particularly true for patients receiving CAR T‐cell therapy. First‐line pharmacotherapy for CRS includes the administration of the IL‐6 receptor‐binding monoclonal antibody tocilizumab, or tocilizumab and corticosteroids. Other agents, such as siltuximab, anakinra, and dasatinab are also being explored for refractory cases of CRS. This review summarizes the potential role of omega‐3 fatty acids, that is, eicosapentaenoic acid (EPA) and docosahexaenoic acid (DHA) at ameliorating CRS in cancer patients receiving immune‐modulating chemotherapy, and is compared with current treatment strategies to reduce the severity of the inflammatory response.

**Recent Findings:**

Selective blockade of specific proinflammatory mediators (e.g., IL‐6) is effective, but carries a significant risk of serious opportunistic infections. In contrast, omega‐3 fatty acids affect multiple triggers underlying the inflammatory response (i.e., prostaglandins, leukotrienes, transcription factors, and specialized proresolving molecules), and its major limitation is avoidance of hypertriglyceridemia, which can be managed by reducing the rate of intravenous administration. This discussion proposes a novel approach by continuous infusion of omega‐3 fatty acids to modulate the intensity of the severe systemic inflammatory response from CRS. The purpose of this review is to highlight the potential clinical benefits of a specialized omega‐3 fatty acids dosage form to mitigate the severity of CRS as a hypothetical alternative to current treatment.

**Conclusion:**

Optimizing the formulation, for example, enriched fish oil that meets drug concentration standards for EPA and DHA, a continuous infusion rate, reductions in long‐chain saturated fatty acids concentrations, and addition of medium‐chain triglycerides to improve EPA + DHA utilization and physical stability are key pharmaceutical factors. This may result in a safer and more effective option than targeted abrogation of cytokines and consequent risks of adverse drug reactions, but will require formal study in randomized control trials in humans.

## 1 Introduction

Cytokine release syndrome (CRS) has been described as a *noninfectious* toxicity induced by cancer immunotherapies [1], which differs from cytokine storm (CS) that arises from highly *infectious*, proinflammatory stimuli, such as in severe COVID‐19 viral illness [2]. No matter the stimulus, a severe and potentially lethal systemic inflammatory response is produced which may be amenable to anti‐inflammatory therapy. According to the National Cancer Institute (NCI) at the US National Institute of Health (NIH), CRS is defined as follows:

A condition that may occur after treatment with some types of immunotherapy, such as monoclonal antibodies and CAR T cells. Cytokine release syndrome is caused by a large, rapid release of cytokines into the blood from immune cells affected by the immunotherapy. Cytokines are immune substances that have many different actions in the body. Signs and symptoms of cytokine release syndrome include fever, nausea, headache, rash, rapid heartbeat, low blood pressure, and trouble breathing. Most patients have a mild reaction, but sometimes, the reaction may be severe or life threatening [3].

Current therapy to mitigate the hyperinflammation that often accompanies immune‐modulating chemotherapy therapy focuses on specific inhibition of selected cytokines, for example, interleukin receptor antagonism, that is, IL‐6 (e.g., tocilizumab) and IL‐1 (anakinra) blockers. However, caution has been advised when employing agents that selectively block cytokine signaling, as they may impair clearance of SARS‐CoV‐2 in severe COVID‐19 infection, increasing the risk of secondary infections [2], which may be pertinent to immune‐modulating therapy. Alternatively, omega‐3 fatty acids (mainly eicosapentaenoic acid or EPA and docosahexaenoic acid or DHA) have wide‐ranging effects on inflammation by altering eicosanoid metabolism as the preferred substrate for cyclooxygenase and lipoxygenase enzymes over omega‐6 fatty acids [4]. This action consequently reduces the intensity of the systemic inflammatory response by disruption of lipid rafts, inhibition of activation of proinflammatory transcription nuclear factor kappa B, activation of the anti‐inflammatory transcription factor NR1C3 and binding to the G‐protein coupled receptor GPR120 [5, 6], as well as the production of specialized proresolving mediators [7, 8]. Hence, given the multi‐targeted actions of omega‐3 fatty acids to reducing the inflammatory response, they may be a safer and more effective option than targeted abrogation of cytokines with the potential for subsequent adverse drug reactions.

## 2 Cytokine Release Syndrome

Of the available FDA‐approved cancer immunotherapies, for example, Chimeric Antigen Receptor (CAR) T‐cell therapy, immune checkpoint inhibitors, and bispecific T‐cell engagers, CAR T‐cell therapy is unique. The patient's T‐cells are harvested (via leukapheresis) and then genetically altered (via CAR‐encoding DNA/RNA) to express a chimeric antigen receptor specifically targeted against certain cancers. This action is followed by ex vivo cell expansion to a predetermined concentration that allows the administration of a dose ranging from 0.2 to 2 million cells/kg of body weight (depending upon the CAR T therapy) in the final formulation that is reinfused into the patient [9, 10]. Consequently, the “final formulation” is considered a “living drug” [11, 12] when compared with conventional “nonliving therapeutics” [13]. Of the approved cancer immunotherapies, CAR T‐cell therapy is a very promising treatment for multiple malignancies and is being actively pursued in various stages of development. It is, however, also a labor‐intensive, patient‐specific process requiring substantial financial investment and resources [14, 15]. Prior to CAR T‐cell therapy, a lymphodepleting chemotherapy regimen is given beforehand, generally consisting of a combination of fludarabine and cyclosphosphamide. After 2 or more days following lymphodepletion therapy, CAR T‐cell therapy commences. The CAR T‐cell platform has significantly developed since its inception 30 years ago, and five generations later the quality, stability, and clinical efficacy of the CAR T‐cell platform has significantly improved with, for example, development of novel intracellular signaling domains [1] and dose modification strategies [16]. Finally, “Tumor Load or Burden” is an important factor affecting clinical outcome, and a high tumor load is often less responsive to immunotherapy, requiring higher doses, and consequently, a higher incidence of adverse reactions.

An acute potentially life‐threatening systemic inflammatory response, such as CRS, can occur with the infusion of bispecific T‐cell engagers, but most significantly with CAR T‐cell therapy [17]. In fact, all of the FDA approved cancer immunotherapies for these two classes contain a “black box” warning in the package insert for life‐threatening CRS following treatment. The official definition of a black box warning can be summed up as follows: “A prominently displayed boxed warning, the so‐called black box, is added to the labeling of drugs or drug products by the Food and Drug Administration when serious adverse reactions or special problems occur, particularly those that may lead to death or serious injury [18].” Moreover, the development of CRS is associated with adverse cardiac effects [19] and has been reported to occur within 7 days of receiving CAR T‐cell therapy [20], with new onset atrial fibrillation being most prevalent [21].

The severity of CRS is based on a typical ranking system from mild (Grade 1) to severe (Grade 4), but harmonization has been lacking. To address this shortcoming, the American Society for Transplantation and Cellular Therapy (ASTCT) proposed harmonized definitions and grading for CRS not attributable to any other cause [22], and has been subsequently endorsed by others [23]. It can be summarized as follows:

**Grade 1**: Fever (≥ 38.0°C) without hypotension or hypoxia.

**Grade 2**: Fever (≥ 38.0°C) with hypotension not requiring vasopressors; hypoxia requiring low‐flow nasal cannula (≤ 6 L/min).

**Grade 3**: Fever (≥ 3 8.0°C) with hypotension requiring 1 vasopressor with or without vasopressin and/or hypoxia requiring high‐flow nasal cannula (≥ 6 L/min).

**Grade 4**: Fever (≥ 38.0°C) with hypotension requiring multiple vasopressors (excluding vasopressin), and hypoxia requiring positive pressure (e.g., CPAP, BiPAP, intubation, and mechanical ventilation).

Tumor load has been shown to be significantly correlated with the severity and incidence of CRS [24], and continued investigative efforts to reduce it are recommended [25]. For example, bridging strategies, defined “as any therapy [e.g., ifosfamide/carboplatin/etoposide] received between leukapheresis and start of lymphodepletion chemotherapy” [26], are designed to reduce tumor burden and improve clinical outcomes. Recently, although in vivo CAR19 expansion is associated with CRS toxicity, it did not correlate with efficacy, suggesting that progression‐free survival should be matched with tumor burden [27].

## 3 Current Treatment Options

According to the FDA‐approved package inserts regarding “CRS Grading and Management Guidance”, the first‐line pharmacological treatment for CRS is with the IL‐6 receptor antagonist, tocilizumab, or tocilizumab and corticosteroids. Pharmacological treatment is even recommended in cases of CRS Grade 1 in patients receiving CAR T‐cell therapy with persistent (> 3 days) or refractory fever. Of note, the use of corticosteroids in the treatment of CRS is associated with increased risk of infection [28]. Alternative agents for treating CRS include another IL‐6 antagonist, such as siltuximab, and the IL‐1 receptor antagonist anakinra, but these are still under investigation [29]. Given the severity and subsequent rapidity (2–3 days) with which CRS can develop and last for 7–8 days [30], there is a tendency to intervene sooner [23], but such an approach for pre‐treatment remains experimental at this time [31]. Moreover, these treatments are also associated with severe adverse drug reactions, as described in the product labeling, such as serious opportunistic infections (tocilizumab, corticosteroids), gastrointestinal perforation (siltuximab), and anaphylactic reactions (anakinra). Finally, caution has been advised when employing agents that selectively block cytokine signaling, as they may impair clearance of SARS‐CoV‐2 in severe COVID‐19 infection, increasing the risk of secondary infections [2], which may be pertinent to CAR T‐cell therapy. Given the limitations in efficacy of current treatment for CRS and association with severe adverse drug effects, as well as the timing for initiation of anti‐inflammatory therapy, alternative therapeutic agents and drug regimens are needed.

## 4 Novel Treatment Options: Potential Mitigating Role of Parenteral Omega‐3 Fatty Acids

A more promising and diverse approach in treating CRS from immune‐modulating chemotherapy may be possible with the parenteral administration of the omega‐3 fatty acids EPA (20:5n3) and DHA (22:6n3). Rather than selective abrogation of certain cytokines, omega‐3 fatty acids influence multiple aspects of the systemic inflammatory response affecting, for example, cytokine secretion (e.g., IL‐1, TNF, interferons), which are fueled by endogenous lipid mediators, including prostaglandins and leukotrienes. Given the high intake of proinflammatory omega‐6 fats and particularly linoleic acid in the normal western diet, compared with the limited dietary intake of anti‐inflammatory omega‐3 fats, the innate immune response is “primed” for hyperinflammation. That is, the long‐chain (mainly 18‐carbons) polyunsaturated omega‐6 linoleic acid (18:2n6) in the diet is elongated to 20 carbon arachidonic acid and then used to form highly proinflammatory prostaglandins of the “2‐series” and leukotrienes of the “4‐series.” In contrast, very long chain (mainly 20 and 22‐carbons) omega‐3 fatty acids (also the *preferred* lipid fuel for cells) form the much less inflammatory “3‐series” prostaglandins and “5‐series” leukotrienes, as well as being the source of specialized proresolving lipid mediators (SPM's) that counteract the inflammatory response and serve to promote recovery [32].

Historically, intravenous natural (unenriched) fish oil emulsion has been used as an alternative source of calories and fatty acids, often used in addition to plant‐based injectable emulsions for over 25 years for critically ill patients receiving parenteral nutrition support. Significant clinical benefits, including reductions in clinical risk of infection, shortened ICU stay, and overall hospital length of stay, have been observed [33, 34]. Of note however, many of these studies used doses of omega‐3 fats that are in the higher range of usual dietary intake, but not to pharmacologic levels as we propose that have at least been shown experimentally to dramatically reduce interleukin‐1 and tumor necrosis factor in response to stimulation in normal humans [35].

The intravenous administration of omega‐3 fatty acid triglycerides greatly accelerates the achievement of stable therapeutic blood levels within hours [36], compared with delivery by enteral (tube‐fed) administration or by oral intermittent supplementation, which can take 7 days [37] to 6 weeks [35], respectively. In the case of CRS, prompt treatment is required for this life‐threatening condition. Such therapy could be considered both as a stand‐alone therapy, or used in conjunction with anticytokine agents, and after experimental study might be useful to be provided prophylactically. Importantly, the intravenous administration of omega‐3 fatty acid triglycerides must be carefully undertaken to avoid profound hypertriglyceridemia. As the hydrocarbon chain length of the infused triglycerides increases, for example, 8–10 carbon fatty acids as medium‐chain triglycerides (MCTs) versus 16–18 carbon vegetable oil‐based long‐chain triglycerides (LCTs) vs. 20‐22‐carbon fish oil‐based LCTs, the clearance from plasma is greatly reduced [38], with also improved clearance for mono‐ and polyunsaturated fatty acids over saturated fatty acids of similar chain length. In a 1994 Editorial, two experts identified the upper infusion rate for mainly 18‐carbon fatty acids derived from soybean oil injectable emulsion administered to be at 0.11 g/kg/h [39] to avoid adverse reactions (e.g., hypertriglyceridemia and reticuloendothelial system dysfunction) [40]. The subsequent “fat overload” syndrome that can occur at higher rates most likely arises when the lipid infusion rate exceeds the Km for lipoprotein lipase for these very LCTs. Natural fish oil contains significant amounts of saturated fatty acids, depending on whether the fish oil is one that is refined (compliant with European Monograph 1912), or one that is both refined and enriched (compliant with European Monograph 1352), according to the processing of the natural fish oil [41]. Enriched fish oil typically doubles the EPA + DHA concentrations relative to natural fish oil (i.e., 22% → 45% EPA + DHA, expressed as triglycerides, in a ratio of 1.5:1) with a concurrent reduction in, for example, long‐chain saturated fatty acids which are also proinflammatory, and this reduction may be a factor as well. Thus, reducing the concentrations of long‐chain saturated fatty acid through enrichment may also improve plasma clearance of infused triglycerides. In fact, when the aforementioned LCT soybean oil triglyceride infusion rate was applied to a refined‐only, very long‐chain fish oil triglyceride injectable emulsion, profound hypertriglyceridemia (> 500 mg/dL) occurred [42].

Moreover, the refined‐only fish oil (EP 1912) is only indicated as a nutritional supplement, and therefore has a very high variability in the concentrations of EPA + DHA (±50%) from batch‐to‐batch. Provision of pharmacological doses of omega‐3 fatty acids (dosed as the sum of EPA + DHA) for severe systemic inflammatory response have been suggested to be in the range of 4–6 g/day [43]. These amounts are logarithmically higher than usual dietary amounts and therefore require a refined and enriched fish oil source that complies with EP 1352. For drug indications where EPA + DHA are the active pharmaceutical ingredient(s) or API, the final concentration must be controlled, for example, (±10%) [32]. Nonetheless, to deliver these very long‐chain fatty acid triglyceride doses from fish oil safely, a 50% reduction in the aforementioned maximum infusion rate applied to soybean oil‐based injectable emulsions [39] has been recommended [44].

There is evidence of the safety profile of up to 6 g of EPA + DHA/day given by intravenous administration in critically ill patients as part of a parenteral nutrition regimen. For example, in a study of 661 critically ill medical and surgical ICU patients (i.e., comprising 531 patients with major abdominal surgery or peritonitis and abdominal sepsis), Heller et al. [45] provided a 100% unenriched fish oil emulsion dosed at 0.1–0.2 g/kg/day for 8.7 ± 7.5 days. A significant reduction in hospital mortality was observed, when compared with SAPS (simplified acute physiology score) II score prediction (18.9% vs. 11.9%, *p < 0.001*). In a similar study of 127 surgical ICU patients undergoing major abdominal surgery, they received an enriched fish oil source contained in a mixed‐oil (50%_w/v_ soybean oil + 40% MCT_w/v_ + 10% _w/v_ enriched fish oil) injectable emulsion as part of a parenteral nutrition regimen in lipid doses of 0.7–1.4 g/kg/day postoperatively for 5 days [46]. A significantly shorter length of hospital stay was observed compared with those who received a soybean oil emulsion as part of the parenteral nutrition regimen (*n* = 129) (17.2 vs. 21.9 days, *p = 0.006*). These outcomes reflect a significant reduction in systemic inflammation, and in both studies, the fish oil emulsions were well tolerated and of note, the body mass index of the patients was similar (BMI ~ 25). The estimated dose of EPA + DHA (g/day) in the Heller et al. study [45] for a 70 kg patient would be approximately 4.2 g/day, whereas in the Wichmann et al. study [46] it would be approximately 5.9 g/day. Importantly, these patients were critically ill and received pharmacological doses of EPA + DHA intravenously which were well tolerated and supports its likely safety in the proposed application for CRS.

Therefore, by devising an optimized pharmaceutical formulation, which uniquely specifies the sum of EPA + DHA as the API that meets pharmacopeial standards as required by the FDA for any drug, provided by continuous intravenous administration to achieve steady state plasma concentrations, it could be safely applied in patients with CRS who are receiving immune‐modulating chemotherapy. Although these characteristics have not been formally tested in humans, a potential first attempt at possible infusion rates that would provide these conditions is suggested. Based on the limited studies to date, the determination of the optimal infusion rates for proposed effects will need to be made after formal clinical study. Finally, given the influence of tumor burden as a precipitating factor in the pathogenesis of CRS, and as a first approximation, we suggest consideration of two tiers of daily doses of EPA + DHA, which would be administered by continuous intravenous infusion, as shown in Table 1.

**TABLE 1:** Twenty‐four hour continuous infusion of a 20%_w/v_ FO/MCT injectable acid emulsion^a^ based on tumor load.

	“Low” tumor load	“High” tumor load
Weight, kg	Tier‐1 dose^b^: 0.03 g/kg/day	Tier‐2 dose^c^: 0.06 g/kg/day
		Daily dose: EPA + DHA, g
	Daily dose: EPA + DHA, g	
10	0.3	0.6
20	0.6	1.2
30	0.9	1.8
40	1.2	2.4
50	1.5	3.0
60	1.8	3.6
70	2.1	4.2
80	2.4	4.8
90	2.7	5.4
100	3.0	6.0

^a^ Refined and enriched fish oil/medium chain triglycerides in a 9:1 FO/MCT ratio providing 100 mg/mL of EPA + DHA.

^b^ Tier‐1 dose based on “low” tumor load.

^c^ Tier‐2 dose based on “high” tumor load.

## 5 Conclusions

Omega‐3 fatty acids have broad anti‐inflammatory properties, affecting a range of proinflammatory mediators involved in CRS. As the preferred substrate for eicosanoid metabolism [4], therapeutic levels of omega‐3 fatty acids can be rapidly achieved by intravenous infusion. For example, during the infusion of an emulsion mixture containing MCT and fish oil (MCT:FO), incorporation into plasma cell membranes of leukocytes and platelets was achieved within 60 min of infusion [36]. The inclusion of MCTs in the formulation along with LCTs not only facilitates plasma clearance of the emulsion by retaining a favorable interfacial location for efficient hydrolysis of the MCT‐LCT lipid droplets [47], but it also significantly improves the physical stability of these thermodynamically unstable lipid injectable emulsions [48]. Therefore, the clinical application of such a formulation, enriched in omega‐3 fatty acids, that can be safely infused, may also be effective in mitigating the toxic effects of CRS during immune‐modulating chemotherapy and should be considered for evaluation in a robust randomized clinical trial against tocilizumab.

## Author Contributions

B.R.B. conceived therapeutic intervention of Ω‐3 fatty acids in pharmacological doses for critically ill patients with severe COVID‐19 infection. D.F.D. defined the requirements for a pharmaceutical formulation that meets pharmacopeial standards to deliver a safe and effective formulation for infusion. B.R.B. and D.F.D. contributed to the critical discussion. D.F.D. drafted the manuscript and B.R.B. edited the manuscript. B.R.B. and D.F.D. reviewed and approved the final version of the manuscript.

## Conflicts of Interest

D.F.D. is the inventor of US Patent No. US 8241672 entitled “Omega‐3 enriched fish oil‐in‐water parenteral nutrition emulsions,” filed on March 11, 2009. B.R.B. is the co‐inventor of US Patent No. 10328045 B2 entitled “Dietary emulsion formulations and methods for using the same,” filed on September 10, 2015.

## Data Availability

The data that support the findings of this study are available in [repository name] at [URL/DOI], reference number [reference number]. These data were derived from the following resources available in the public domain: [list resources and URLs].
